# Multiplex PCR for simultaneous genotyping of *kdr* mutations V410L, V1016I and F1534C in *Aedes aegypti* (L.)

**DOI:** 10.1186/s13071-020-04193-0

**Published:** 2020-06-25

**Authors:** Karina Villanueva-Segura, Gustavo Ponce-Garcia, Beatriz Lopez-Monroy, Esteban Mora-Jasso, Lucia Perales, Francisco J. Gonzalez-Santillan, Kevin Ontiveros-Zapata, Jesus A. Davila-Barboza, Adriana E. Flores

**Affiliations:** Universidad Autonoma de Nuevo Leon (UANL), Av, Universidad s/n Cd. Universitaria, 66455 San Nicolas de los Garza, N.L. Mexico

**Keywords:** Multiplex PCR, *Aedes aegypti*, *kdr*, V410L, V1016I, F1534C

## Abstract

**Background:**

Knockdown resistance (*kdr*) is the main mechanism that confers resistance to pyrethroids and DDT. This is a product of non-synonymous mutations in the voltage-gated sodium channel (*vgsc*) gene, and these mutations produce a change of a single amino acid which reduces the affinity of the target site for the insecticide molecule. In Mexico, V410L, V1016I and F1534C mutations are common in pyrethroid-resistant *Aedes aegypti* (L.) populations.

**Methods:**

A multiplex PCR was developed to detect the V410L, V1016I and F1534C mutations in *Ae. aegypti*. The validation of the technique was carried out by DNA sequencing using field populations previously characterized for the three mutations through allele-specific PCR (AS-PCR) and with different levels of genotypic frequencies.

**Results:**

The standardized protocol for multiplex end-point PCR was highly effective in detecting 15 genotypes considering the three mutations V410L, V1106I and F1534C, in 12 field populations of *Ae. aegypti* from Mexico. A complete concordance with AS-PCR and DNA sequencing was found for the simultaneous detection of the three *kdr* mutations.

**Conclusions:**

Our diagnostic method is highly effective for the simultaneous detection of V410L, V1016I and F1534C, when they co-occur. This technique represents a viable alternative to complement and strengthen current monitoring and resistance management strategies against *Ae. aegypti*.
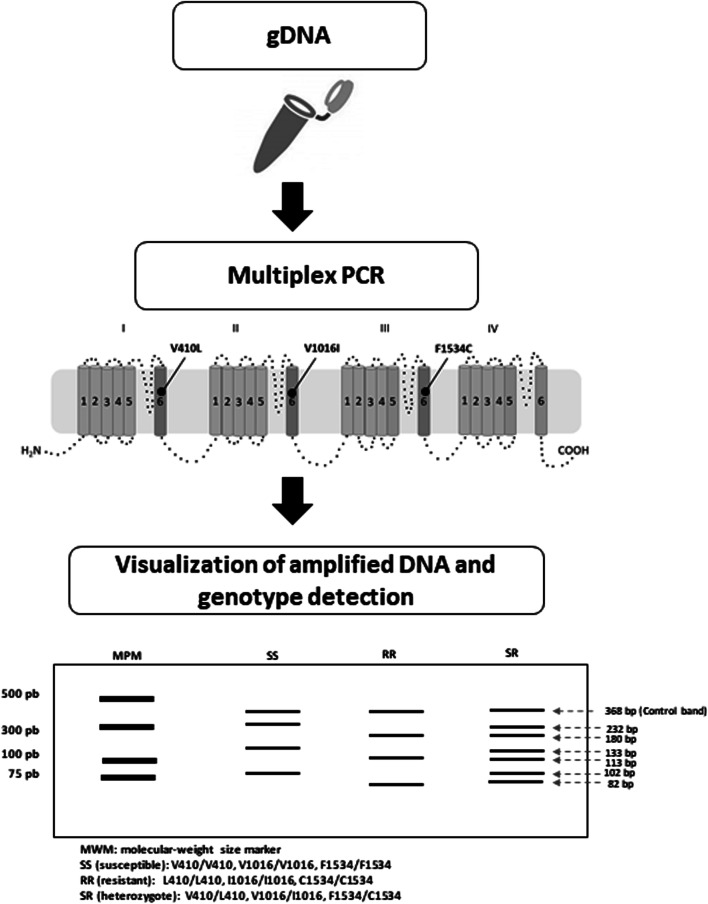

## Background

The portfolio of insecticides available for the control of arthropod pathogen vectors is very limited and is unlikely to increase in the near future, mainly due to the high cost of developing new molecules and products. Therefore, the emergence of resistance to commonly used insecticides is a serious threat to our ability to fight diseases transmitted by *Aedes aegypti* (L.). The development of such resistance is a complex and dynamic process dependent on many factors, so its management requires two types of information: good knowledge of the resistance mechanisms and their monitoring. The characterization of the resistance mechanisms involved allows us to assess and predict their impact on vector control strategies. Having routine monitoring of insecticide resistance in natural vector populations helps us to detect early resistance and improve the effectiveness of operational control strategies. *Aedes aegypti* is the main vector of dengue, chikungunya and Zika virus in Mexico, and its control depends largely on the use of pesticides that vary in their mode of action and include organophosphates, carbamates and neonicotinoids, but pyrethroids remain the preferred class, due to their fast action, high insecticidal activity and low toxicity to mammals [1, 2]. The target site of pyrethroids is the *vgsc* present in the axon membrane of neurons and excitable cells in insects, and these insecticides produce a knockdown effect, that is, instantaneous paralysis in the insect due to prolonged activation and subsequent blockage of the action potentials of these channels [1, 3–5].

The main mechanisms that confer resistance to pyrethroids are overexpression of detoxifying enzymes and/or insensitivity at the target active site, being the mechanism associated with detoxification enzymes identified in *Ae. aegypti* populations from different regions of Mexico, particularly the mechanisms associated with esterases, glutathione S-transferases and mixed-function oxidases [6–9]. On the other hand, knockdown resistance (*kdr*) is conferred principally by nonsynonymous mutations that reduce pyrethroid binding to *vgsc*. In Mexico, it has been determined that pyrethroid resistance in *Ae. aegypti* is associated with high frequencies of any of the V1016I, F1534C and V410L mutations or combinations thereof. V1016I was the first to be reported in a population of *Ae. aegypti* from Isla Mujeres, Quintana Roo, Mexico resistant to permethrin [10]. Subsequently, Ponce-Garcia et al. [11] demonstrated through a retrospective analysis carried out with 78 collections of *Ae. aegypti* that this replacement was practically absent in samples collected between 1996 and 2001, showing a dramatic increase between 2007 and 2009. Additionally, Siller et al. [12] reported an increase in allele I1016 frequency in *Ae. aegypti* populations in 2009 in several locations in Mexico. Vera-Maloof et al. [13] performed a linkage disequilibrium analysis in populations collected in Mexico between 2000 and 2012 that carried I1016 and C1534, and their results suggested that the sequential evolution of both mutations was necessary for pyrethroid resistance to develop. Saavedra-Rodriguez et al. [14] reported the V410L mutation in *Ae. aegypti* collections obtained during 2002 to 2016 from several locations in Mexico, observing a high frequency of V410L in collections previously genotyped with V1016I and F1534C. Later, Villanueva-Segura et al. [15] reported high allelic frequencies of L410 in 25 populations of *Ae. aegypti* occurring in 2018 in eastern and southern Mexico with frequencies of 0.10–0.99.

Currently, there are different PCR-based techniques for the detection of *kdr* mutations, which offer high sensitivity and specificity. However, when choosing one of these techniques, it is important to consider the economic resources of the laboratory, the training of technical personnel and the time available [16]. This point is even more important when considering the geographical extent that the vector occupies, the quantity of samples to be processed and the dynamics and fluctuation of the *kdr* resistance in short periods of time. The high-performance techniques available for genotyping *kdr* mutations, which are characterized by their speed and high sensitivity, are: sequencing of specific regions of *vgsc*, real-time PCR with TaqMan probes and high-resolution melting (HRM). However, given the need to acquire specialized equipment and supplies and the quantity of samples to be processed, the high cost of these methods constitutes its greatest disadvantage. The above has made allele specific PCR (AS-PCR) one of the most used techniques for this purpose, offering a low cost and low error rate. Despite this, for laboratories in developing or low-budget countries, the use of other techniques based on AS-PCR is still recommended, when considering cost and ease of use and performance [17–20]. Recently, the multiplex PCR technique has proven effective in the identification of two *kdr* mutations (V1016G and F1534C) simultaneously in *Ae. aegypti* [21]. In addition, it is possible to adapt it to different biochemical assays that not only reveal the amplified products optimally, but also contribute to improving the reliability, cost and performance of resistance monitoring [22].

The aim of this study was to develop a multiplex PCR method to detect the three mutations, V410L, V1016I and F1534C, reported in *Ae. aegypti* in Mexico, in a single reaction. This technique can reduce the cost and time spent to monitor allelic frequencies in many countries where all three mutations co-occur.

## Methods

### Mosquito collections

Field collections of *Ae. aegypti* were carried out in 2018 from six states in Mexico: Nuevo Leon in the northeast with two locations (Monterrey and Guadalupe); Veracruz in the southeast with four locations (Poza Rica, Minatitlan, Cardel and Cosoleacaque); also in the southeast Tabasco with one location (Villa Hermosa); Chiapas in the south with one location (Tapachula); Yucatan in the southeast with three locations (Merida, San Antonio Kaua and Vergel); and Quintana Roo with one location (Cancun).

Mosquitoes collected in the field were at immature stages, and they were reared to adults under laboratory conditions at 25 ± 4 °C and a 12:12 h L:D photoperiod. They were morphologically identified and stored at − 20 °C until DNA extraction.

Pyrethroid resistance was confirmed with bottle bioassays for permethrin (DD 15 µg/bottle) and deltamethrin (10 µg/bottle) [23] with mortalities ranging from 3 to 87% for permethrin and 58 to 86% for deltamethrin.

### DNA isolation

DNA was isolated from ~30 individual mosquitoes per location using the salt extraction technique and resuspended in 50 µl of ultrapure molecular grade water (Corning Cellgro^TM^, Manassas, VA, USA) [24]. The concentration and quality of each DNA sample was determined on a NanoDrop 2000 spectrophotometer (Thermo Fisher Scientific, Woonsocket, RI, USA).

### Development of the multiplex PCR method

The amplification primers used for the variants of loci 410, 1016 and 1534 are given in Additional file 1: Table S1.

The specific oligonucleotides L410f and 410r amplify a region of 113 bp, corresponding to the L410 allele (resistant). The specific oligonucleotides V410f and 410r amplify a region of 133 bp, corresponding to the V410 allele (susceptible). The specific oligonucleotides I1016f and I1016r amplify a region of 82 bp, corresponding to the I1016 allele (resistant). Oligonucleotides V1016f and I1016r amplify a region of 102 bp, corresponding to the V1016 allele (susceptible). The oligonucleotides c1534-f and c1534-r amplify a region of 368 bp with which the specific oligonucleotides Ae1534F-r and Ae1534C-f hybridize, resulting in products of 180 bp for the C1534 allele (resistant) and of 232 bp for the F1534 allele (susceptible) (Additional file 2: Figure S1).

Tests for optimization of PCR conditions resulted in the following multiplex PCR protocol. The DNA samples used in the amplification process were in a concentration range of 20–250 ng/µl. The final reaction mixture was 19.12 µl and contained: 1.02× buffer (Invitrogen, Carlsbad, CA, USA), 1.53 mM MgCl_2_, 0.2 mM dNTPs, oligonucleotides for genotyping 410 at a final reaction concentration of 1.27 pmol/µl, for 1016 at 1.02 pmol/µl and for 1534 at 0.82 pmol/µl (Additional file 1: Table S1), and also 5 U *Taq* DNA polymerase.

The reaction was carried out in a Multigene Optimax thermal cycler (Labnet International, Edison, NJ, USA). The reaction conditions were as follows: 95 °C for 2 min for the initial separation of the DNA strands, followed by 45 cycles of 95 °C (30 s), 58.6 °C (1 min) and 72 °C (30 s) and a final extension for 2 min.

A PCR tube containing all the components except genomic DNA was run with the primers as a contamination control. Controls were included in each PCR performed, the New Orleans strain was used as susceptible control.

After amplification, 4 µl of the products of the PCR reaction mixture were analyzed by horizontal electrophoresis on a 2.5% agarose gel. The electrophoresis conditions were 110 V for 1 h using 1× SB buffer (200 mM sodium borate buffer, pH 8) along with a 25 bp molecular weight marker to determine the size of the fragments and staining with GelRed® (Biotium, Hayward CA, USA). The PCR products were visualized with a transilluminator (UVITEC, Cambridge, UK). At the end of amplification, it was possible to obtain up to 7 PCR products per sample, whose size indicated the genotypic combination for loci 410, 1016 and 1534.

### Validation of the study

#### AS-PCR

To validate the results obtained in multiplex PCR, AS-PCR was performed according to Saavedra et al. [10], Yanola et al. [25] and Villanueva-Segura et al. [15].

#### Sequencing

Sequences of primers to detect *vgsc* point mutations were according to Saavedra et al. [14] for domain IS6 (exon 9-10), Kushwah et al. [26] for domain IIS6 (exon 20-21) and Chung et al. [27] for domain IIIS6 (exon 31). A 200 bp segment at domain I was amplified with the primers V410f (5′-GCG GGC AGG GCG GCG GGG GCG GGG CCA TCT TCT TGG GTT CGT TCT ACC GTG-3′), L410f (5′-GCG GGC ATC TTC TTG GGT TCG TTC TAC CAT T-3′) and L410r (5′-TTC TTC CTC GGC GGC CTC TT-3′); a 350 bp segment at domain II was amplified with the primers AedIIF (5′-AGA CAA TGT GGA TCG CTT CC-3′) and AedIIR (5′-GGA CGC AAT CTG GCT TGT TA-3′); and the 700 bp segment at domain III was amplified with primers AaSCF7 (5′-GAG AAC TCG CCG ATG AAC TT-3′) and AaSCR7 (5′-GAC GAC GAA ATC GAA CAG GT-3′). The polymerase chain reaction was carried out using GoTaq® DNA Polymerase Master Mix with 1.0, 0.5 and 0.4 μM of each primer for domains I6, II6 and III6, respectively and 100 ng of DNA as a template. PCRs were performed under the following temperature programs: 94 °C for 3 min; 35 cycles of 94 °C for 15 s; 56.1 °C (domain I6), 60 °C (domain II6) and 55 °C (domain III6) as annealing temperature and 1 min elongation time at 72 °C followed by 5 min at 72 °C. The amplicons were visualized by gel red staining after electrophoresis in 2% agarose gels and sent for sequencing to Macrogen Corporation (Rockville, MD, USA). The sequences were aligned using BioEdit Sequence Alignment Editor 7.0.5.3 and analyzed in MEGA version 7 [28, 29].

## Results

### Multiplex PCR *vs* AS-PCR assay

**Fig. 1:**
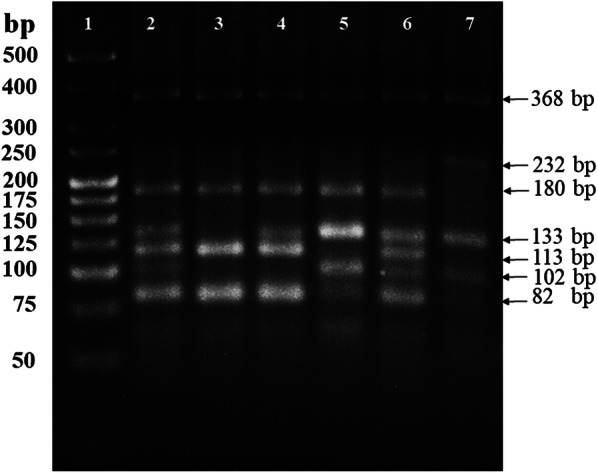
Agarose gel (2.5%) electrophoresis of amplified gDNA products in several mosquitoes using multiplex PCR. The arrows indicate a common band of 368 bp, a band of 232 bp corresponding to the susceptible allele F1534, a band of 180 bp corresponding to the resistant allele C1534, a band of 133 bp corresponding to the susceptible allele V410, a band of 113 bp corresponding to the resistant allele L410, a band of 102 bp corresponding to the susceptible allele V1016, and a band of 82 bp corresponding to the resistant allele I1016. Lane 1: molecular weight marker (25 bp DNA Ladder); Lane 2: a resistant homozygous (C1534/C1534), heterozygous (V410/L410) and heterozygous (V1016/I1016) individual; Lane 3: a resistant homozygous individual (C1534/C1534), resistant homozygous (L410/L410) and resistant homozygous (I1016/I1016); Lane 4: a resistant homozygous individual (C1534/C1534), heterozygous (V410/L410) and resistant homozygous (I1016/I1016); Lane 5: a resistant homozygous individual (C1534/C1534), susceptible homozygous (V410/V410) and heterozygous (V1016/I1016); Lane 6: a resistant homozygous individual (C1534/C1534), heterozygous (V410/L410) and heterozygous (V1016/I1016); Lane 7: a triple susceptible homozygous individual (V410/V410, V1016/V1016 and F1534/F1534)

Mosquito DNA of each population was used to detect the mutations V410L, V1016I and F1534C. Molecular assays were conducted on 352 mosquitoes of the populations analyzed. The genotype L410/L410 (homozygous mutant) was seen as a single band of 113 bp and the homozygous wild-type genotype (V410/V410) as a single band of 133 bp, while the heterozygous genotype (V410/L410) showed both bands. Genotype I1016/I1016 (homozygous mutant) was seen as a single band of 82 bp and the homozygous wild-type genotype (V1016/V1016) as a single band of 102 bp, while the heterozygous genotype (V1016/I1016) showed both bands. The homozygous wild-type genotype (F1534/F1534) showed a single band of 232 bp and the homozygous mutant genotype (C1534/C1534) a single band of 180 bp, while the heterozygous genotype (F1534/C1534) had both bands. In the case of the detection of the F1534C mutation, an internal control band of 368 bp is obtained (Fig. 1).

A total of 15 genotypes of the 27 possible were obtained and validated in the populations analyzed, using multiplex PCR and the individual end-point PCR technique previously reported by Saavedra et al. [10], Yanola et al. [25] and Villanueva-Segura et al. [15] (Table 1).

**Table 1:** Comparison of genotyping results for V410L, V1016I, F1534C mutations from multiplex PCR, AS-PCR and DNA sequencing

Population	Multiplex PCR / AS-PCR/ DNA sequencing															
	LL/II/CC	LL/VI/CC	LL/VV/CC	LL/VI/FC	VL/II/CC	VL/VI/CC	VV/II/CC	VV/VI/CC	VL/VI/FC	LL/VV/FF	VV/VI/FC	VV/VV/CC	VL/VV/CC	VL/VV/FC	LL/II/FC	*n*
Monterrey	1/1/1	6/6/6	4/4/4	0/0/0	2/2/2	9/9/9	1/1/1	1/1/1	0/0/0	0/0/0	0/0/0	4/4/4	0/0/0	0/0/0	0/0/0	28
Guadalupe	5/5/5	6/6/6	0/0/0	1/1/1	4/4/4	10/10/10	1/1/1	3/3/3	0/0/0	0/0/0	0/0/0	0/0/0	0/0/0	0/0/0	0/0/0	30
Poza Rica	0/0/0	2/2/2	17/17/17	0/0/0	0/0/0	5/5/5	2/2/2	0/0/0	0/0/0	0/0/0	0/0/0	1/1/1	2/2/2	0/0/0	0/0/0	29
Minatitlan	17/17/17	0/0/0	0/0/0	1/1/1	0/0/0	1/1/1	0/0/0	0/0/0	0/0/0	1/1/1	0/0/0	0/0/0	0/0/0	0/0/0	10/10/10	30
Cardel	26/26/26	1/1/1	0/0/0	0/0/0	1/1/1	2/2/2	0/0/0	0/0/0	0/0/0	0/0/0	0/0/0	0/0/0	0/0/0	0/0/0	0/0/0	30
Cosoleacaque	4/4/4	0/0/0	0/0/0	0/0/0	2/2/2	10/10/10	0/0/0	0/0/0	1/1/1	0/0/0	9/9/9	0/0/0	0/0/0	1/1/1	0/0/0	27
Villa Hermosa	15/15/15	0/0/0	0/0/0	0/0/0	0/0/0	14/14/14	0/0/0	0/0/0	0/0/0	0/0/0	0/0/0	1/1/1	0/0/0	0/0/0	0/0/0	30
Chiapas	1/1/1	4/4/4	0/0/0	1/1/1	2/2/2	14/14/14	0/0/0	1/1/1	4/4/4	0/0/0	1/1/1	0/0/0	1/1/1	0/0/0	0/0/0	29
Merida	9/9/9	0/0/0	0/0/0	0/0/0	1/1/1	16/16/16	0/0/0	1/1/1	0/0/0	0/0/0	0/0/0	2/2/2	1/1/1	0/0/0	0/0/0	30
San Antonio Kaua	15/15/15	0/0/0	0/0/0	0/0/0	1/1/1	9/9/9	0/0/0	3/3/3	0/0/0	0/0/0	0/0/0	2/2/2	0/0/0	0/0/0	0/0/0	30
Vergel	9/9/9	1/1/1	0/0/0	0/0/0	0/0/0	19/19/19	0/0/0	0/0/0	0/0/0	0/0/0	0/0/0	1/1/1	0/0/0	0/0/0	0/0/0	30
Cancun	3/3/3	0/0/0	0/0/0	0/0/0	7/7/7	6/6/6	6/6/6	5/5/5	0/0/0	0/0/0	1/1/1	0/0/0	0/0/0	0/0/0	1/1/1	29

*Abbreviations*: VV, wild type (susceptible); VL, heterozygotes; LL, mutant (resistant); VI, heterozygotes; II, mutant (resistant); FF, wild type (susceptible); FC, heterozygotes; CC, mutant (resistant); *n* sample size

### Multiplex PCR *vs* DNA sequencing

The sequences of all samples were also in agreement with the multiplex PCR demonstrating that all samples had the same genotype using multiplex PCR as with AS-PCR and sequenced (Table 1). The sequences of L410/L410, I1016/I1016, C1534/C1534 have been deposited in GenBank under the accession numbers MT233424 (L410/L410), MT250049 (I1016/I1016) and MT250050 (C1534/C1534).

The analysis of the frequencies of the L410, I1016 and C1534 was determined in the 352 mosquitoes of the 12 selected populations. The frequencies for the allele L410 ranged from 0.36 in the population of Cancun from Quintana Roo and 0.98 in the population of Minatitlan from Veracruz. Allelic frequencies for I1016 ranged from 0.19 in the Poza Rica population to 0.97 in the Minatitlan population, both from the state of Veracruz. The lowest frequency for the allele C1534 was observed coincidently in the Minatitlan population in which the highest frequency of the alleles L410 and I1016 was obtained. The mutation F1534C was fixed in 7 of the 12 populations analyzed (Table 2).

**Table 2:** Comparison of genotypes for V410L, V1016I and F1534C mutations for multiplex PCR, AS-PCR and DNA sequencing

Population	*n*	Multiplex PCR / AS-PCR/ DNA sequencing											
		410			Frequency	1016			Frequency	1534			Frequency
		VV	VL	LL	L410	VV	VI	II	I1016	FF	FC	CC	C1534
Monterrey	28	6/6/6	11/11/11	11/11/11	0.59	8/8/8	16/16/16	4/4/4	0.43	0/0/0	0/0/0	28/28/28	1.0
Guadalupe	30	4/4/4	14/14/14	12/12/12	0.63	0/0/0	20/20/20	10/10/10	0.67	0/0/0	1/1/1	29/29/29	0.98
Poza Rica	29	3/3/3	7/7/7	19/19/19	0.78	20/20/20	7/7/7	2/2/2	0.19	0/0/0	0/0/0	29/29/29	1.0
Minatitlan	30	0/0/0	1/1/1	29/29/29	0.98	1/1/1	2/2/2	27/27/27	0.97	1/1/1	11/11/11	18/18/18	0.78
Cardel	30	0/0/0	3/3/3	27/27/27	0.95	0/0/0	3/3/3	27/27/27	0.95	0/0/0	0/0/0	30/30/30	1.0
Cosoleacaque	27	10/10/10	13/13/13	4/4/4	0.39	1/1/1	20/20/20	6/6/6	0.59	0/0/0	10/10/10	17/17/17	0.81
Villa Hermosa	30	1/1/1	14/14/14	15/15/15	0.73	1/1/1	15/15/15	14/14/14	0.72	0/0/0	0/0/0	30/30/30	1.0
Chiapas	29	2/2/2	21/21/21	6/6/6	0.57	1/1/1	25/25/25	3/3/3	0.53	0/0/0	6/6/6	23/23/23	0.90
Merida	30	3/3/3	18/18/18	9/9/9	0.60	3/3/3	17/17/17	10/10/10	0.62	0/0/0	0/0/0	30/30/30	1.0
San Antonio Kaua	30	5/5/5	10/10/10	15/15/15	0.67	2/2/2	12/12/12	16/16/16	0.73	0/0/0	0/0/0	30/30/30	1.0
Vergel	30	1/1/1	19/19/19	10/10/10	0.65	1/1/1	20/20/20	9/9/9	0.63	0/0/0	0/0/0	30/30/30	1.0
Cancun	29	12/12/12	13/13/13	4/4/4	0.36	0/0/0	12/12/12	17/17/17	0.79	0/0/0	2/2/2	27/27/27	0.97

*Abbreviations*: VV, wild type (susceptible); VL, heterozygotes; LL, mutant (resistant); VI, heterozygotes; II, mutant (resistant); FF, wild type (susceptible); FC, heterozygotes; CC, mutant (resistant); *n* sample size

In the populations analyzed, the most frequent genotype was double heterozygous for 410 and 1016 and homozygous resistant for 1534 (V410/L410 + V1016/I1016 + C1534/C1534) followed by the triple homozygous resistant (L410/L410 + I1016/1016 + C1534 + C1534). The least frequent genotypes were homozygous resistant for 410, and double wild type for 1106 and 1534 (L410/L410 + V1016/V1016 + F1534/F1534), and double heterozygous for 410 and 1534 and wild type for 1016 (V410/L410 + V1016/V1016 + F1534/C1534) (Table 1).

## Discussion

Performing PCR even today still takes time and effort; however, the objective of developing a multiplex PCR method is mainly to reduce these factors by being able to amplify various alleles in the same reaction. This is difficult when it comes to standardizing the method, where each pair of primers included increases the difficulty, since it is not enough to match the Tm (melting temperature) and AG (adenosine:guanidine) of the primers, therefore extra effort is needed in their design [30].

The optimization of every multiplex PCR method has critical difficulties. The design of the primers is key to a successful PCR, and the presence of more than one pair increases the possibility of dimers and also requires the adjustment of the other PCR components (buffers, dNTPs, MgCl_2_ and *Taq* DNA polymerase) [31].

One of the main utilities of multiplex PCR is the simultaneous detection of multiple genes, such as serogroups of pathogens like with *Salmonella*, *Escherichia coli* O157 and *Listeria monocytogenes* [32, 33], including allele-specific multiplex PCR for detection of multi-resistance in *Mycobacterium* tuberculosis [34]. However, the utility of multiplex PCR is not limited to this; one of the first reports of detection of multiple *kdr* mutations for the 1014 site by multiplex PCR was performed by Tan et al. [35] for the alleles L/S (TTG/TCG), L/F (TTG/TTT) and L/L (TTG/TTG). Assays have been performed for the detection of V1016I/G and F1534C mutations individually [10, 25, 36], and as multiplex PCR with other mutations such as V1016G and F1534C, T1520I and F1534C [21, 26]. The V410L mutation was first detected in co-occurrence with the V1016I and F1534C mutations in *Ae. aegypti* mosquitoes but only by simplex PCR [14].

The utility of multiplex PCR is also not limited only to the detection of a single resistance mechanism. Kazanidou et al. [37] standardized the detection of polymorphisms by the substitution of five nucleotides in the sodium channel gene and the *ace-1* gene ([*kdr*-w homozygous], [*kdr*-e homozygous], [*kdr* heterozygous], *ace-1r* homozygous, and their hybrids [*ace1s*/*ace-1r*, *kdr*s/*kdr*-w]) in only one of the samples.

In this study, we used a simplex PCR and DNA genotyping as confirmation techniques to characterize each mutation in the samples used and to validate the multiplex PCR method. Twelve samples were used among which we could determine up to 15 different genotypes. The results of the test validation showed 100% agreement between multiplex PCR, simplex PCR and DNA sequencing. These results indicated high sensitivity and specificity for the new multiplex PCR method developed. This method allows us to determine the frequency of multiple mutations, as well as their co-occurrence, in a single reaction.

Our method is the first designed for multiplex genotyping of the three *kdr* mutations in *Ae. aegypti* in Mexico, V410L, V1016I and F1534C.

## Conclusions

The multiplex PCR technique allows simultaneous and reliable detection of the V410L, V1016I and F1534C mutations in *Ae. aegypti* populations in Mexico. This method optimizes the monitoring times of mutant alleles and genotypic frequencies in field populations of this species. The characteristics of this technique make it an advantageous alternative for countries where the dynamics of pyrethroid resistance in mosquitoes is variable and changing and where resources for this purpose are also limited.

## Supplementary information

**Additional file 1: Table S1.** Sequences of the primers used for multiplex PCR.

**Additional file 2: Figure S1.** Schematic of the AS-PCR assay for detection of the V410L (**a**), V1016I (**b**) and F1534C (**c**) mutations.

## Data Availability

All data generated or analyzed during this study are included in this published article and its additional files. The newly generated sequences were deposited in the GenBank database under the Accession Numbers MT233424, MT250049 and MT250050.

## Abbreviations

*kdr*: knockdown resistance
*vgsc*: voltage gated sodium channel
AS-PCR: allele-specific PCR
HRM: high-resolution melting

## References

[1] Carvalho FD, Moreira LA (2017). Why is *Aedes aegypti* Linnaeus so successful as a species?. Neotrop Entomol..

[2] Secretaria de Salud. Lista de productos recomendados por el CENAPRECE para el combate de insectos vectores de enfermedades a partir de 2019. https://www.gob.mx/cms/uploads/attachment/file/469289/Lista_de_Insumos_Recomendados_por_el_CENAPRECE.pdf. Accessed 10 Nov 2019.

[3] Narahashi T, Ginsburg KS, Nagata K, Song JH, Tatebayashi H (1998). Ion channels as targets for insecticides. Neurotoxicology..

[4] Dong K, Du Y, Rinkevich F, Nomura Y, Xu P, Wang L (2014). Molecular biology of insect sodium channels and pyrethroid resistance. Insect Biochem Mol..

[5] Silver KS, Du Y, Nomura Y, Oliveira EE, Salgado VL, Zhorov BS (2014). Voltage-gated sodium channels as insecticide targets. Adv Insect Physiol..

[6] Alvarez LC, Ponce G, Saavedra-Rodriguez K, Lopez B, Flores AE (2015). Frequency of V1016I and F1534C mutations in the voltage-gated sodium channel gene in *Aedes aegypti* in Venezuela. Pest Manag Sci..

[7] Flores AE, Albeldaño-Vazquez W, Fernandez-Salas I, Badii MH, Loaiza Becerra H, Ponce Garcia G (2005). Elevated a-esterases levels associated with permethrin tolerance in *Aedes aegypti* (L.) from Baja California, Mexico. Pest Biochem Phys..

[8] Flores AE, Reyes G, Fernandez-Salas I, Sanchez FJ, Ponce G (2009). Resistance to permethrin in *Aedes aegypti* (L.) in northern Mexico. Southwest Entomol..

[9] Flores AE, Ponce G, Silva BG, Gutierrez SM, Bobadilla C, Lopez B (2003). Wide spread cross resistance to pyrethroids in *Aedes aegypti* (L.) from Veracruz State Mexico. J Econ Entomol..

[10] Saavedra-Rodriguez K, Urdaneta-Marquez L, Rajatileka S, Moulton M, Flores AE, Fernandez-Salas I (2007). A mutation in the voltage-gated sodium channel gene associated with pyrethroid resistance in Latin America *Aedes aegypti*. Insect Mol Biol..

[11] Ponce-Garcia G, Flores AE, Fernandez-Salas I, Saavedra-Rodriguez K, Reyes-Solis G, Lozano-Fuentes S (2009). Recent rapid rise of a permethrin knock down resistance allele in *Aedes aegypti* in Mexico. PLoS Neglect Trop Dis..

[12] Siller Q, Ponce G, Lozano S, Flores AE (2011). Update on the frequency of Ile1016 mutation in voltage-gated sodium channel gene of *Aedes aegypti* in Mexico. J Am Mosq Cont Assoc..

[13] Vera-Maloof FZ, Saavedra-Rodriguez K, Elizondo-Quiroga AE, Lozano-Fuentes S, Black WC (2015). Coevolution of the Ile1016 and Cys1534 mutations in the voltage gated sodium channel gene of *Aedes aegypti* in Mexico. Plos Neglect Trop Dis..

[14] Saavedra-Rodriguez K, Maloof FZ, Campbell CL, Garcia-Rejon J, Lenhart A, Penilla P (2018). Parallel evolution of *vgsc* mutations at domains IS6, IIS6 and IIIS6 in pyrethroid resistant *Aedes aegypti* from Mexico. Sci Rep..

[15] Villanueva-Segura K, Ontiveros-Zapata K, Lopez-Monroy B, Ponce-Garcia G, Gutierrez-Rodriguez S, Davila-Barboza JA (2020). Distribution and frequency of the *kdr* mutation V410L in natural populations of *Aedes aegypti* (L.) (Diptera: Culicidae) from eastern and southern Mexico. J Med Entomol..

[16] Martins AJ, Valle D, Soloneski S, Larramendy ML (2012). The pyrethroid knockdown resistance. Insecticides—basic and other applications.

[17] Kolaczinski JH, Fanello C, Herve JP, Conway DJ, Carnevale P, Curtis CF (2000). Experimental and molecular genetic analysis of the impact of pyrethroid and non-pyrethroid insecticide impregnated bednets for mosquito control in an area of pyrethroid resistance. Bull Entomol Res..

[18] Lynd A, Ranson H, McCall PJ, Randle NP, Black WC, Walker ED (2005). A simplified high-throughput method for pyrethroid knock-down resistance (*kdr*) detection in *Anopheles gambiae*. Malar J..

[19] Kulkarni MA, Rowland M, Alifrangis M, Mosha FW, Matowo J, Malima R (2006). Occurrence of the leucine-to-phenylalanine knockdown resistance (*kdr*) mutation in *Anopheles arabiensis* populations in Tanzania, detected by a simplified high-throughput SSOP-ELISA method. Malar J.

[20] Bass C, Nikou D, Donnelly MJ, Williamson MS, Ranson H, Ball A (2007). Detection of knockdown resistance (*kdr*) mutations in *Anopheles gambiae*: a comparison of two new high-throughput assays with existing methods. Malar J.

[21] Saingamsook J, Saeung A, Yanola J, Lumjuan N, Walton C, Somboon P (2017). A multiplex PCR for detection of knockdown resistance mutations, V1016G and F1534C, in pyrethroid-resistant *Aedes aegypti*. Parasit Vectors..

[22] Henry-Halldin CN, Nadesakumaran K, Keven JB, Zimmerman AM, Siba P, Mueller I (2012). Multiplex assay for species identification and monitoring of insecticide resistance in *Anopheles punctulatus* group populations of Papua New Guinea. Am J Trop Med Hyg..

[23] Brogdon W, Chan A. Guidelines for evaluating insecticide resistance in vectors using the CDC bottle bioassay. CDC technical report. Methods in *Anopheles* research. 2nd edn. Atlanta: Centers for Disease Control and Prevention; 2010.

[24] Coen E, Strachan T, Dover G (1982). Dynamics of concerted evolution of ribosomal DNA and histone gene families in the melanogaster species subgroup of *Drosophila*. J Mol Biol..

[25] Yanola J, Somboon P, Walton C, Nachaiwieng W, Somwang P, Prapanthadara LA (2011). High-throughput assays for detection of the F1534C mutation in the voltage-gated sodium channel gene in permethrin-resistant *Aedes aegypti* and the distribution of this mutation throughout Thailand. Trop Med Int Health..

[26] Kushwah R, Dykes C, Kapoor N, Adak T, Singh O (2015). Pyrethroid-resistance and presence of two knockdown resistance (*kdr*) mutations, F1534C and a novel mutation T1520I, in Indian *Aedes aegypti*. PLoS Neglect Trop Dis..

[27] Chung HH, Cheng IC, Chen YC, Lin C, Tomita T, Teng J (2019). Voltage-gated sodium channel intron polymorphism and four mutations comprise six haplotypes in an *Aedes aegypti* population in Taiwan. PLos Neglect Trop Dis..

[28] Hall TA (1999). BioEdit: a user-friendly biological sequence alignment editor and analysis program for Windows 95/98/NT. Nucl Acids Symp Ser..

[29] Kumar S, Stecher G, Tamura K (2016). MEGA7: molecular evolutionary genetics analysis version 7.0 for bigger datasets. Mol Biol Evol..

[30] Alvarez-Fernandez R (2013). Explanatory chapter: PCR primer design. Method Enzymol..

[31] Hernandez-Cortez C, Mendez-Tenorio A, Aguilera-Arreola MG, Castro-Escarpulli G (2013). Design and standardization of four multiplex polymerase chain reactions to detect bacteria that cause gastrointestinal diseases. Afr J Microbiol Res..

[32] Lavalett L, Sanchez MM, Muñoz N, Moreno J, Cardona-Castro N (2009). Desarrollo y validacion de una reacción en cadena de la polimerasa multiple para la identificacion de los serogrupos B, C2, D y E de *Salmonella enterica*. Biomedica..

[33] Garrido A, Chapela MJ, Roman B, Fajardo P, Vieites JM, Cabado AG (2013). In-house validation of a multiplex real-time PCR method for simultaneous detection of *Salmonella* spp., *Escherichia coli* O157 and *Listeria monocytogenes*. Int J Food Microbiol..

[34] Bing-Shao C, Lanzas F, Rifat D, Herrera A, Kim EY, Sailer C (2012). Use of multiplex allele-specific polymerase chain reaction (MAS-PCR) to detect multidrug-resistant tuberculosis in Panama. PLoS One..

[35] Tan WL, Li CX, Wang ZM, Liu MD, Dong YD, Feng XY (2012). First detection of multiple knockdown resistance (*kdr*)-like mutations in voltage-gated sodium channel using three new genotyping methods in *Anopheles sinensis* from Guangxi Province, China. J Med Entomol..

[36] Fernando SD, Hapugoda M, Perera R, Saavedra-Rodriguez K, Black WC, De Silva NK (2018). First report of V1016G and S989P knockdown resistant (*kdr*) mutations in pyrethroid-resistant Sri Lankan *Aedes aegypti* mosquitoes. Parasit Vectors..

[37] Kazanidou A, Nikou D, Grigoriou M, Vontas J, Skavdis J (2009). Short report: a multiplex PCR assay for simultaneous genotyping of *kdr* and *ace-1* loci in *Anopheles gambiae*. Am J Trop Med Hyg..

