# Supplementary material for: Multiplex PCR for simultaneous genotyping of kdr mutations V410L, V1016I and F1534C in Aedes aegypti (L.)
Source: Parasit Vectors. 2020 Jun 25;13:325. doi: 10.1186/s13071-020-04193-0 (PMC7318494; doi:10.1186/s13071-020-04193-0)
Supplement: Supplementary file 1 — Additional file 1: Table S1. Sequences of the primers used for multiplex PCR. [file 13071_2020_4193_MOESM1_ESM.docx]

**Additional file 1: Table S1.** Sequences of the primers used for multiplex PCR.

| **Primer name** | **5’-3’ Sequence** | **Reference** |
| --- | --- | --- |
| 410 Genotyping |  | [14] |
| V410f* | [LongGCTail]ATCTTCTTGGGTTCGTTCTACCGTG |  |
| L410f** | [ShortGCTail]ATCTTCTTGGGTTCGTTCTACCATT |  |
| 410r | TTCTTCCTCGGCGGCCTCTT |  |
|  |  |  |
| 1016 Genotyping |  | [10] |
| V1016f* | [LongGCTail]ACAAATTGTTTCCCACCCGCACCGG |  |
| I1016f** | [ShortGCTail]ACAAATTGTTTCCCACCCGCACTGA |  |
| I1016r | TGATGAACCSGAATTGGACAAAAGC |  |
|  |  |  |
| 1534 Genotyping |  | [21] |
| c1534-f | GCGTACCTGTGTCTGTTCCA |  |
| c1534-r | GGCTTCTTCGAGCCCATCTT |  |
| Ae1534F-r | GCGTGAAGAACGACCCGA |  |
| Ae1534F-f | CCTCTACTTTGTGTTCTTCATCATCTG |  |

*Long Tail 26b GCGGGCAGGGCGGCGGGGGCGGGGCC; **Short tail 6b GCGGGC
