# Supplementary figures and images for: Multiplex PCR for simultaneous genotyping of kdr mutations V410L, V1016I and F1534C in Aedes aegypti (L.)
Source: Parasit Vectors. 2020 Jun 25;13:325. doi: 10.1186/s13071-020-04193-0 (PMC7318494; doi:10.1186/s13071-020-04193-0)

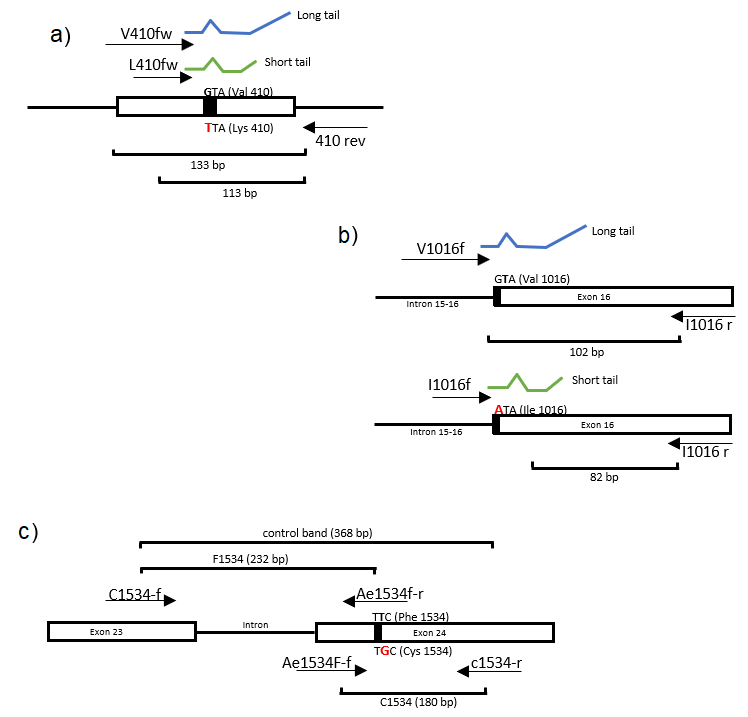

Supplement: Supplementary file 2 — Additional file 2: Figure S1. Schematic of the AS-PCR assay for detection of the V410L (a), V1016I (b) and F1534C (c) mutations. [file 13071_2020_4193_MOESM2_ESM.tif]
